# Precise Diagnosis and Therapy of Bone Cancer Using Near-Infrared Lights

**DOI:** 10.3389/fbioe.2021.771153

**Published:** 2021-11-18

**Authors:** Qing Cai, Zuntai Li, Baosheng Li, Jiayang Jiang, Xiaoyu Li, Weiyan Meng, Shoujun Zhu

**Affiliations:** ^1^ Hospital of Stomatology, Jilin University, Changchun, China; ^2^ State Key Laboratory of Supramolecular Structure and Materials, College of Chemistry, Jilin University, Changchun, China

**Keywords:** bone tumor, osteopathia, near-infrared probe, diagnosis, photoX therapy

## Abstract

Bone is a preferred site for both primary and metastasis tumors. Current diagnosis of osteopathia typically relies on noninvasive skeleton radiography technology. However, due to the limited resolution of ionizing radiation, accurate diagnosis and effective identification impairment areas are still lacking. Near-infrared (NIR) bioimaging, especially in the NIR-II (1000-1700 nm) regions, can provide high sensitivity and spatiotemporal resolution bioimaging compared to the conventional radiography. Thus, NIR bioimaging affords intraoperative visualization and imaging-guided surgery, aiming to overcome challenges associated with theranostics of osteopathia and bone tumors. The present review aimed to summarize the latest evidence on the use of NIR probes for the targeting bone imaging. We further highlight the recent advances in bone photoX (X presents thermal, dynamic, and immuno) therapy through NIR probes, in particular combination with other customized therapeutic agents could provide high-efficiency treatment for bone tumors.

## Introduction

Bone tumors are generally classified into orthotopic tumors and metastatic tumors. Osteosarcoma is the most common orthotopic bone cancer and the third most common cancer among children and adolescents (Simpson and Brown, 2018). Bone is also a preferred site for tumor hematogenous metastasis such as breast, prostate, or lung cancer. There are more than 600,000 cases of bone metastases diagnosed every year in the United States in older adults (>40 years of age) (Krzeszinski and Wan, 2015). Hence, it is necessary to diagnose the disease in an early stage and personalize treatments based on patient’s individual variability (Rubin et al., 2014). Currently, varieties of imaging techniques are used in the clinical practice, including magnetic resonance imaging (MRI), computed tomography (CT), ultrasound (US), positron emission tomography (PET), single-photon emission tomography (SPECT). However, CT and MRI often require high doses of contrast agents; PET and SPECT require radioactive tracers, increasing the safety concern (Mariani et al., 2002; Tsien, 2003). Thus, a noninvasive, accurate, and efficient diagnosis and therapeutic response monitoring of bone cancer are urgently needed in order to meet the needs of the clinician.

Near-infrared (NIR) fluorescence imaging (700–1700 nm), which benefits from minimal tissue absorption, scattering, and auto-fluorescence, is favorable for *in vivo* imaging with a high signal-to-background ratio (SBR) (Teitelbaum, 2000; Gu et al., 2012; Jiang et al., 2012; Zhu et al., 2013; Sun et al., 2016; Zhao et al., 2016; Yang et al., 2017). Compared to traditional diagnosis modalities, NIR fluorescence imaging offers advantages in biosafety, imaging resolution, and speed (Liu et al., 2019). This technique could integrate multiplexing of signals and evaluate interactions between bone-specific molecular targets, the microenvironment, and tumor metastasis (Cho and Shokeen, 2019). Recently, the discovery of NIR-II (1,000–1700 nm) imaging modality further increases the penetration depth and imaging contrast compared with NIR-I (700–1,000 nm) window (Zhu et al., 2018; Zhu S. et al., 2019), providing improved *in vivo* imaging quality for deep tissue visualization (Xu et al., 2021).

There are several unsolved issues in traditional imaging modalities. First, overwhelming binding affinity of the imaging probe results in excessive deposition of the probe in the bone cortex, thus blurring the imaging resolution of cancellous bone (Harrison and Cooper, 2015). This phenomenon can affect the precise assessment of the bone state and fail to diagnose the tiny lesions (Hashimoto et al., 2020). Second, bone disease and other relative diseases may affect the local skeletal condition in terms of bone mineral density, organizational change, and other characteristic diagnostic markers, further increasing the difficulties in disease diagnosis (Armstrong et al., 2018). The collection of per-lesion basis image features is the vital evidence, which can be used to determine lesion-wise response (Yip and Jeraj, 2014). At last, it is still difficult to accurately differentiate between osteogenic and osteoclastic states in the lesion marginal area which is the most desirable information for clinicians (Morimoto et al., 2021). These phenomena hinder the application of imaging diagnosis in clinic. The advent of the NIR fluorescent probe promises to lead bone imaging diagnosis out of the current predicament. This review article starts with the bone physiology and bone tumor microenvironment and then summarizes the synthesis and categories of imaging mechanism of current NIR probes with bone targeting ability. We further discuss the bone cancer diagnosis by NIR probes and photoX therapy of bone cancer by NIR probes. The challenges of bone-targeting NIR probes are also discussed.

## The Bone Physiology and Bone Tumor Microenvironment

Bone is composed of three main cell types, osteoblasts, osteoclasts, and osteocytes, which are responsible for maintaining structure through precise remodeling (Buck and Dumanian, 2012). Osteoblasts, derived from mesenchymal stem cells (MSCs) in the bone marrow, could synthesize and secrete the organic bone matrix. The organic bone matrix is composed of type I collagen (90%), non-collagenous proteins, water, and hydroxyapatite (Shupp et al., 2020). Mature osteoclasts derived from monocytes could solubilize the bone matrix via acidification and also resorb mineralized bone (Clarke, 2008). Osteoclasts bind to the bone matrix via integrin receptors in the osteoclast membrane. Osteoclasts express cathepsin K and other enzymes that aid the acidified resorption of bone (Teitelbaum, 2000). The osteocytes lie in lacunae within the mineralized bone and have extensive filopodial processes in the canaliculi of mineralized bone (Bonewald, 1999). Whereas the earliest functions proposed for osteocytes were mechanosensing and removal of their perilacunar matrix, an unanticipated function was the osteocyte-producing factors that could regulate both bone cells and distant organs (e.g., kidney) (Robling and Bonewald, 2020). Besides cell composition, the primary mineral content of bone is hydroxyapatite [Ca_10_(PO_4_)_6_(OH)_2_], which is approximately 200 Å in their largest dimension. The calcium- and phosphate-binding proteins, including osteocalcin, osteopontin, and bone sialoprotein, contribute to regulate hydroxyapatite crystallization and ordered deposition of minerals (Clarke, 2008).

The bone microenvironment is composed of bone marrow and a mineralized extracellular matrix (Yang et al., 2020). The most exceptional aspect of the bone metastasis biology is that the developmental sites include the host hematopoietically active red marrow (Kricun, 1985) and the subversion of the osteolysis and osteogenesis processes (Mundy, 2002). Nonetheless, the tumor microenvironment (TME) is much more complicated than bone microenvironment, including molecular elements, signaling pathways, and mechanical properties (Mbeunkui and Johann, 2009). In addition, the bone marrow MSCs in the tumor microenvironment help tumor cells evade the immune attack by avoiding the immune recognition and instigating an immunosuppressive TME (Comşa et al., 2012; Gonzalez et al., 2018). Biological signals contribute to the pathogenesis of cancer (Molina et al., 2020), while physical factors (tissue architecture, matrix stiffness) change phenotypes of cancer cells (Rice et al., 2017). Collectively, much less is known on the molecular mechanism of bone TME. Investigating these parameters is essential to understanding the disease and guiding the development of future therapeutic strategies.

## Fluorescence Probes With Targeting Bone Characteristic

### Synthesis

There are 50–70% inorganic substances in bone, among which hydroxyapatite (HA) is the main component of inorganic minerals in bone tissue and is also the most important target for bone fluorescence imaging. Anionic ligands such as phosphate- and carboxylate-rich compounds were designed to chelate to the Ca^2+^ of bone. Different targeting groups have different ligands, which were conjugated with fluorescence dyes to endow these molecules with bone-targeting properties (Gao et al., 2020).

**FIGURE 1:**
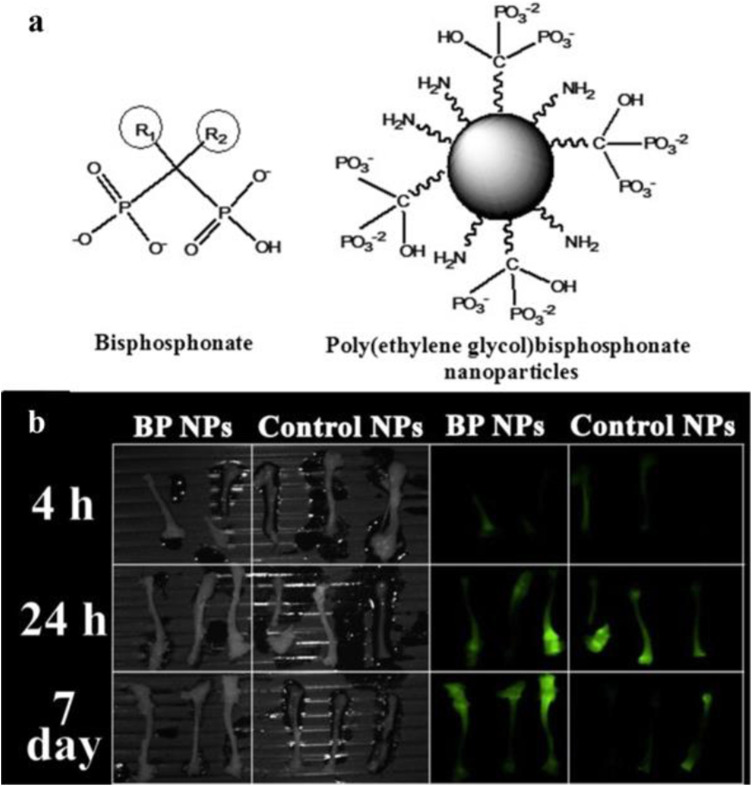
Nanoparticles with bisphosphonates (BPs) functional groups could target bone. **(A)** Chemical structure of BP and poly(ethylene) BP nanoparticles. **(B)** NIR fluorescence imaging of the bones extracted from mice treated with Cy7-labeled BP NPs. Reproduced with permission from Rudnick-Glick et al. (2015).

Bisphosphonates (BPs) and analogs are widely used bone-targeting ligands. Due to their high affinity to Ca^2+^ ions, BPs could rapidly localize into bone minerals (Russell, 2011). The P-C-P moiety is responsible for the strong affinity between BPs and hydroxyapatite through tridentate-binding sites, increasing the resistance of BPs to chemical and enzymatic degradation (Papapoulos, 2008; Russell, 2011). To label bone-binding ligands with NIR probes, the cross-linked BP nanoparticles were covalently conjugated with NHS-activated NIR dye Cy7 (Gluz et al., 2013; Gluz et al., 2014). The BP particles, which enhanced photostability and biocompatibility of the fluorescence dye, possessed higher inhibition activity than alendronate (Gluz et al., 2013) and exhibited affinity to the chicken embryo bones (Gluz et al., 2014) (Figure 1). Further research demonstrated the poly(MA-PEG-BP) NPs prolonged half-life and preferential uptake in areas of bone with high activity (Rudnick-Glick et al., 2015).

Aspartic acid-rich polypeptides, such as Asp_8_ (Wang et al., 2007), Asp_7_ (Jiang et al., 2014), Asp_6_ (Takahashi et al., 2008), and (AspSerSer)_6_ (Zhang et al., 2012), have bone-targeting characteristics. This bone-specific targeting is based on the peptide’s high affinity for hydroxyapatite, which is related to the physicochemical properties of acidic amino acid in polypeptide (Takahashi, 2016). Asp_8_ has a stronger affinity for smaller lattices than mature lattices, which may be related to its folded structure (Wang et al., 2007). In particular, octapeptide Asp_8_ is a commonly used targeting ligand for bone-resorption surface, preferentially for the binding of highly crystallized hydroxyapatite (Wang et al., 2007). The strength of its binding depends on the density of aspartic acid unit, and higher density results in stronger affinity with bone. Although the bone-binding strength of Asp_8_ is relatively weaker than BPs (Wang et al., 2007), the facile synthesis and chemical modification of the Asp_8_ are essential for practical applications. Wang et al. used the Asp_8_ as a bone-targeting ligand to deliver NIR photothermal agents (dendritic platinumcopper alloy nanoparticles) to bone tissues for targeting photothermal therapy (PTT) of bone tumors (Wang et al., 2017). Using the affinities between iminodiacetate and phosphonates for bone minerals, two types of bifunctional contrast agents were recently reported to target bone tissue (Zhang et al., 2020; Liu et al., 2021). In order to modify the physicochemical properties of fluorescence dye, the phosphonates or other affinities were also incorporated into the fluorophore, which promoted the bone-targeting potency and *in vivo* performance (Harmatys et al., 2013; Hyun et al., 2014).

Tetracycline is another hydroxyapatite-targeting compound, while its side effects limited the clinical/laboratory-scale usage (Xie et al., 2018). Currently, tetracycline derivatives are exploited to reduce the side effects of tetracycline. For example, a tetracycline derivative bound to IRDye 800CW (Kovar et al., 2011) and a calcium-chelating tetracycline derivative (IRDye 680RD BoneTag) (Joseph et al., 2014) were introduced to image sites of bone remodeling. Nevertheless, taking account of the side effects, there is no application of tetracycline derivatives in bone tumors.

There are other bone-targeting ligands that can be potentially applied to bone cancer imaging (e.g., succinic acid) (Neale et al., 2009). In addition, a variety of carboxylate-containing ligands or polymers can also deliver the bone-targeting feature to the probes. Nanoparticles modified with nitrodiacetic acid exhibited a strong bone-binding affinity (Wang et al., 2016). The formation of bone-binding moiety depends on the spatial distance between the carboxyl groups, and nitrodiacetic acid has three-fold bone-binding affinity than nitrodipropionic acid (Harmatys et al., 2013). Due to their high bone-binding affinity, biological safety, stability, and hydrophilicity, these bone-targeting groups are extensively used in bone-targeting imaging.

### Targeting Bone Composition and Imaging Quality

**FIGURE 2:**
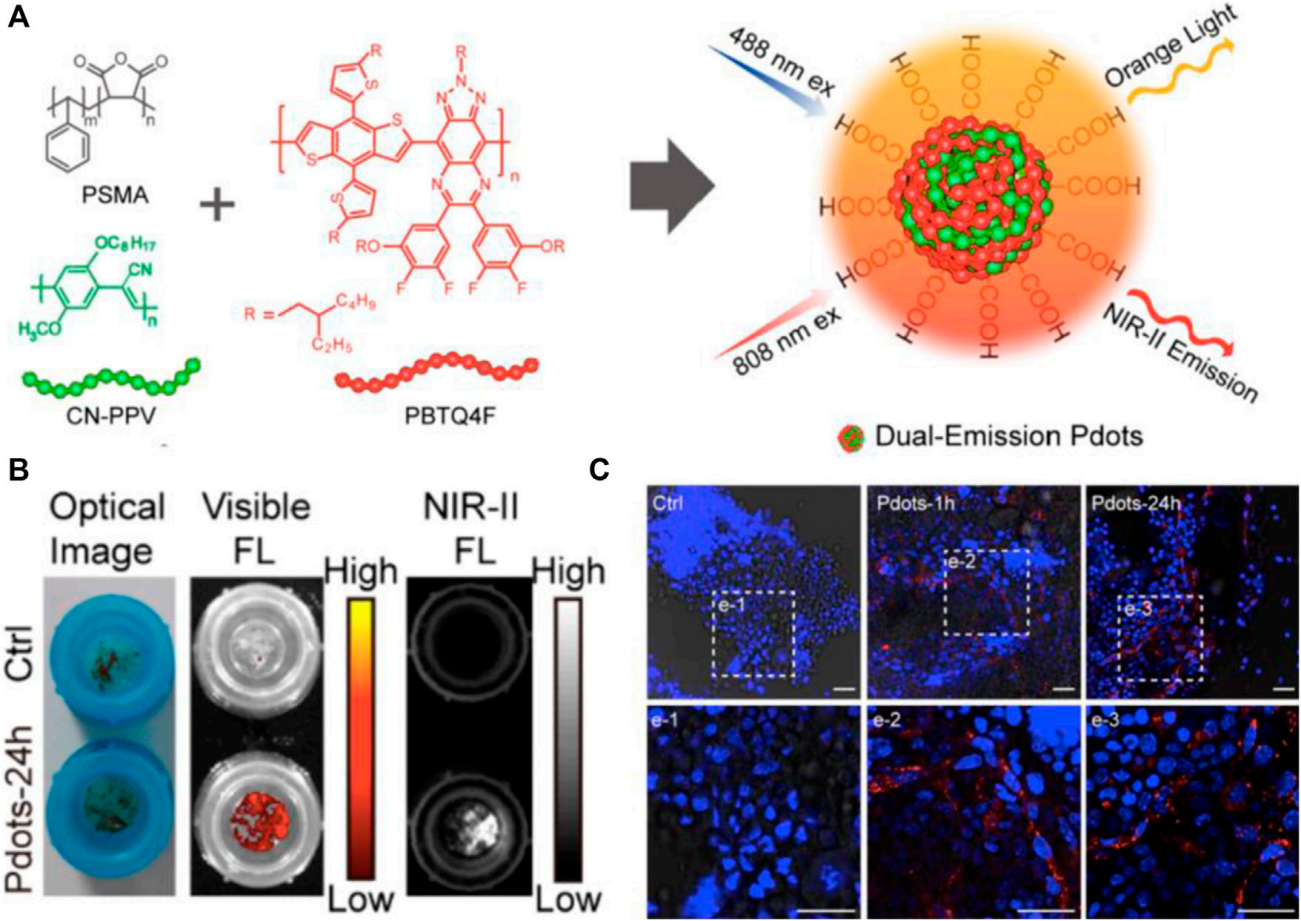
NIR-II fluorescence imaging reveals bone marrow retention using dual-emission polymer dots (Pdots). **(A)** Schematic illustration for the preparation of dual-emission Pdots. **(B)** Optical photograph and fluorescence imaging of bone marrow retained by a cell strainer. **(C)** Confocal images of bone marrow cells retained by a cell strainer. Scale bar: 25 μm. Reproduced with permission from Chen et al. (2021).

For precisely discriminating bone cancer, the designed fluorescent probes should selectively target bone mineral (Li et al., 2017), osteoblast (Lin et al., 2020), osteoclast (Lim et al., 2017), and bone marrow (Chen et al., 2021). Previous studies have proved that the charge and hydrophobicity of targeted fluorophores are essential in the biodistribution, blood circulation, organ accumulation, and clearance route of intravenously administered contrast agents (Bao et al., 2015). Currently, alendronate (Ald) conjugated to the surface of DOX/Ag_2_S QDs, guiding the probe specifically deposited in bone mineral, while inhibiting cancer-associated osteolysis. The NIR-II fluorescence imaging could outline the delivery process of DOX (Li et al., 2017). Rare earth-doped nanoparticles (RENPs) (coated with DSPE-mPEG) showed the distribution in cancellous and compact bone with NIR-II signal, due to the unique hydroxyapatite mineral-binding ability (∼88%). In this process, the circulating leukocytes may promote the NIR-II probes into the bone (He et al., 2019). However, the shortcoming of RENPs is its avoidable accumulation in prejudicial reticuloendothelial system and metabolism pathway *in vivo* like other PEG-encapsulated nanoparticles (Li et al., 2017). Tumor invasion during skeletal metastatic progression shows osteogenetic and osteolytic features. Lin et al. evaluated the conjugation of IR-783 with a synthetic modified α5-binding peptide in the α5β1L-CyTE777 probe to osteoblast cells. The results demonstrated that α5β1L-CyTE777 specifically monitored osteoblastic differentiation (Lin et al., 2020). During the renal carcinoma and breast cancer metastases, the osteolytic lesion is the primary feature. A previous study showed that P800SO3 strongly bounded to active osteoclasts, allowing for visualizing osteolytic lesions under NIR-I fluorescence imaging (Lim et al., 2017). As expected, the location of high osteoclastic activity was clearly marked by the probe in hind limbs and spine of mice, consistent with the histological examination. The tumor cells in bone, especially the metastasis tumor cells, preferentially travel to and colonize the epiphysis of long bones, which has massive bone marrow with a remarkably high rate of bone turnover (Marks and Odgren, 2002; Bussard et al., 2010). The rich microenvironment of bone marrow facilitates survival of cancer cells and mediates drug resistance (Meads et al., 2008). Chen et al. verified that small polymer nanoparticles of ∼15 nm diameter showed fast accumulation and long-term retention in bone. Further results of bone sections by confocal microscopy indicated that poly(styrene-co-maleic anhydride) functionalized polymer dots (Pdots-PSMA) were largely distributed in the endothelial cells of sinusoidal vessels in bone marrow (Chen et al., 2021) (Figure 2). Collectively, the difference of targeting the position of bone between Pdots and RENPs might depend on the diameter of nanoparticles.

Imaging quality is an important indicator to evaluate whether NIR technology can be applied in the clinical setting. With the development of bone-targeting probes, the NIR signal intensity of bone can reach 20-fold than that of surrounding tissues (Kovar et al., 2011). However, early generation of NIR fluorescent probes suffered from nonspecific accumulation in the soft tissues, liver, and intestines (Mizrahi et al., 2011). The newer generation of the bone-targeting probe provided high-performance intraosseous tumor imaging. The fluorescently outlined bone tumor boundary was identical to the histologically confirmed boundary (Lim et al., 2017). The higher binding affinity of bone-targeting probes will afford higher SBR, thus improving the imaging quality (Lin et al., 2020). However, it is difficult for cell-targeting probes to achieve comparable SBR to HA-targeting probes. In the first attempt to image osteocytes, the fluorescence intensity of the targeted cells was only 5–7 times higher than that of the control cells (Cowles et al., 2013). Recent investigation promoted the SBR of the bone cell–targeting probe to 18, which is sufficient to provide valuable diagnosis information (Cho et al., 2021). The current NIR probes have higher sensitivity and resolution than the traditional X-ray and MR imaging modalities, potentially enabling the early diagnosis of intraosseous lesions in clinic (Slooter et al., 2015).

**FIGURE 3:**
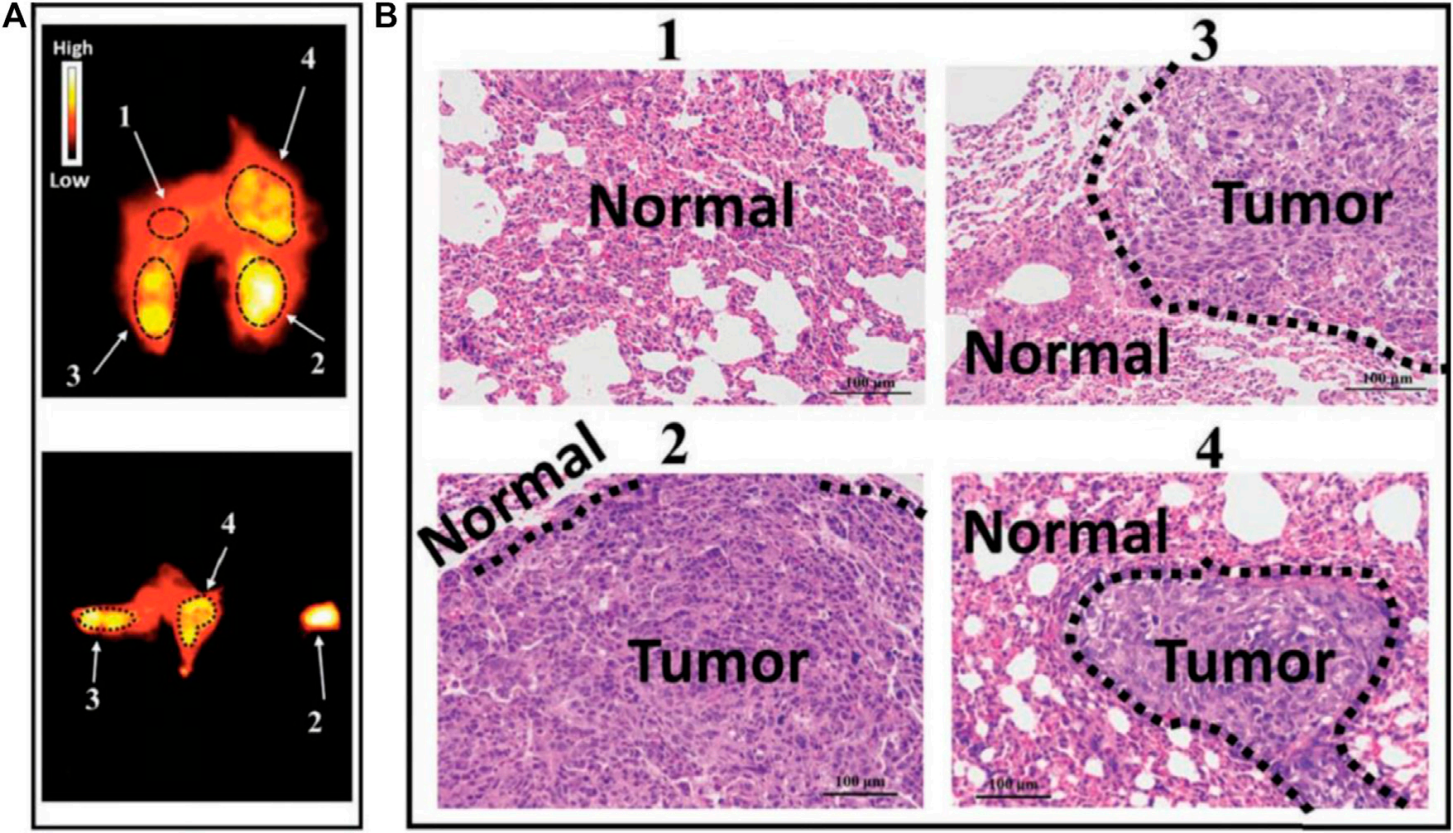
NIR-II imaging of osteosarcoma lung metastasis imaging with high imaging contrast and resolution by CH1055-PEG-Affibody. **(A)** NIR-II fluorescent signals of the lung for lung metastasis evaluation. **(B)** H&E staining results of parts 1, 2, 3, and 4 in the NIR-II lung imaging supported that probe could diagnose small lesions (<1 mm in diameter) that could not be detected by the CT technique. Reproduced with permission from Zhou et al. (2019).

**FIGURE 4:**
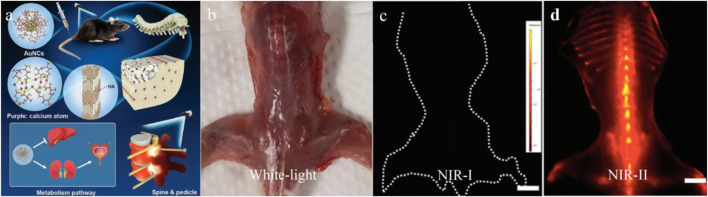
Gold nanoclusters for NIR-II fluorescence imaging of bones. **(A)** The structure of AuNCs and the schematic for bone targeting. **(B–D)** Compared with the clinically available ICG, the AuNCs showed obvious fluorescence in spinal, vertebral vertebrae, distal femur, and proximal tibia. Scale bar: 10 mm. Reproduced with permission from Li et al. (2020).

Although NIR bioimaging has shown remarkable results in bone imagingthe challenge remains that the tissue boundary is not clearly defined (Lim et al., 2020). This phenomenon is partially caused by tissue scattering and limited penetration depth of NIR bioimaging (Cowles et al., 2013). The application of NIR-II fluorescent dye in bone imaging fundamentally overcomes the aforementioned limitations. The SBR of the NIR-II dye administrated tumor model was significantly improved, and tumor lesions less than 1 mm can be clearly detected (Zhou et al., 2020) (Figure 3). This deep penetration depth and high imaging contrast allow NIR-II probes to clearly outline the small indentation in the bone and precisely determine the status of bone osteoporosis in mice, which is more sensitive than the commonly used quantitative computed tomography (QCT) (Zhang et al., 2021). Recently, NIR-II nano-gold probes provided a relatively rapid bone deposition rate and fast excretion feature, enabling an increased penetration depth up to 6 mm (Li et al., 2020) (Figure 4). Together, NIR-II probes with higher imaging quality and penetration depth are promising in bone imaging, bone cancer diagnosis, bone state judgment, etc.

## Bone Cancer Diagnosis by NIR Probes

Bone-related cancers have been divided into primary bone tumor (osteosarcoma, Ewing sarcoma, and chondrosarcoma) and bone metastases (lung tumor, breast cancer, prostatic cancer, etc.) (Riquelme et al., 2020). Currently, it is still challenging to accurately locate and diagnose the bone cancers (Karamzade-Ziarati et al., 2019). The clinician commonly prefers plain radiography to diagnose malignant bone tumors. However, only obviously positive radiographic indicator to malignancy of bone tumors can promote further inspection, usually causing considerable bone damage (Ferguson and Turner, 2018). At the beginning of tumor colonization, tumor cells and the bone marrow microenvironment synergistically regulate tumor growth, mainly including osteoclast activation and slightly dissolve hydroxyapatite (Pang et al., 2020). When the bone-targeting probe was applied as a diagnosis method, tiny changes in bone density could be clearly visualized, providing valuable information for early diagnosis (Slooter et al., 2015). Furthermore, the indistinguishable boundaries of bone tumors force surgeons to strike a balance between reducing positive margin rates and preserving the function of bone (Baljer et al., 2020). In contrast, we hypothesize that future examination of bone tumors will rely on NIR bioimaging, providing accurate tumor margin information in both pre- and intraoperative processes.

**FIGURE 5:**
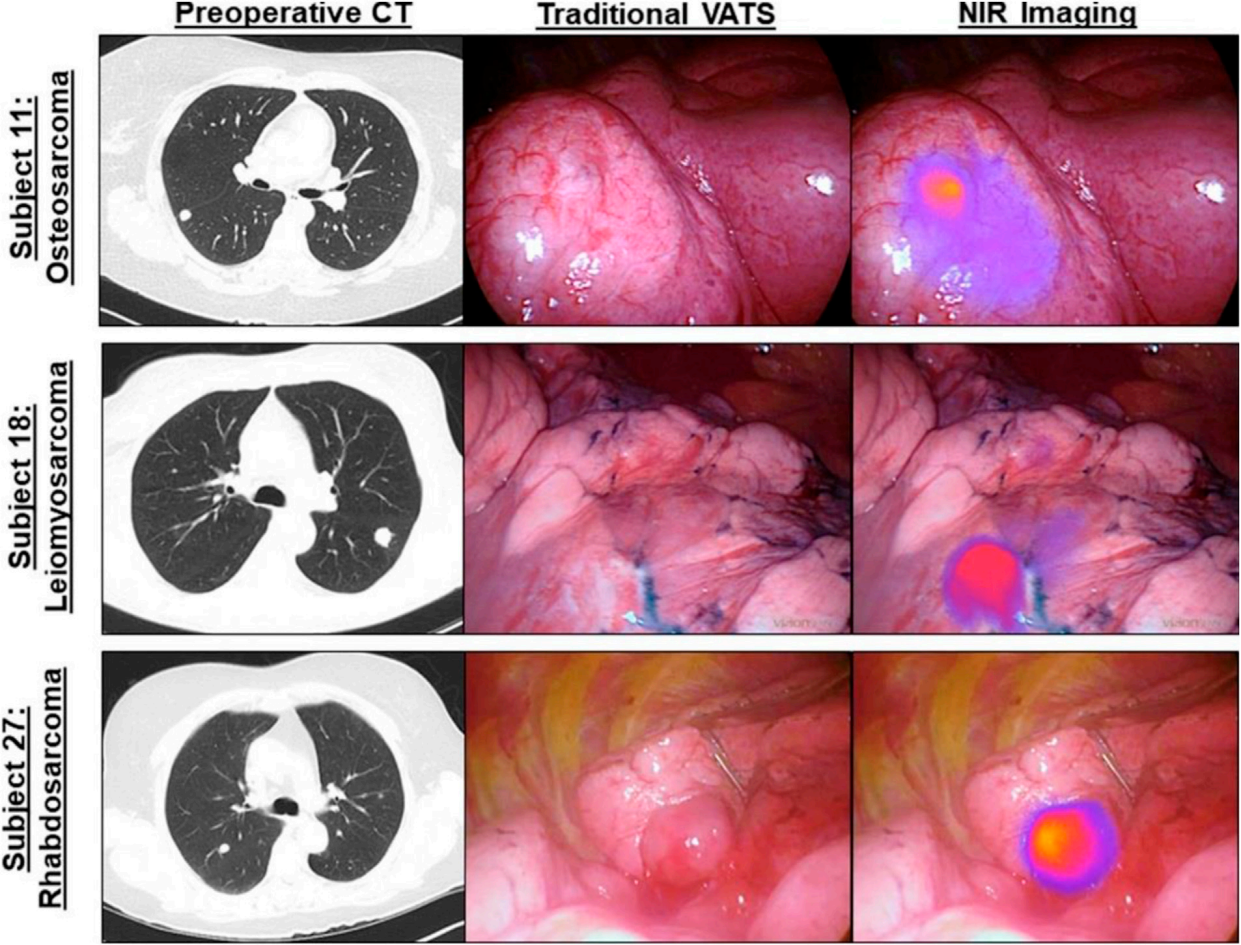
Osteosarcoma metastasis is clearly visualized by NIR intraoperative imaging (ICG) during video-assisted thoracoscopic surgery (VATS) metastasectomy. Left column, preoperative high-resolution computed tomography (CT). Middle column, bright field (traditional views). Right column, NIR merged view. Reproduced with permission from Predina et al. (2018).

The commonest bone cancer is osteosarcoma with rare histologic subtypes, and it is challenging to differentiate the clinicopathologic features with commoner subtypes (Whelan and Davis, 2018). Both non-targeted and targeted NIR bioimaging were applied to visualize the bone tumors. The non-targeted approach benefits bone tumors with abundant blood vessels, and typical examples are primary tumor of knee and partial metastatic tumor (Wuisman and Grunert, 1994). The targeted approach provides potential to distinguish cancer cells from normal tissue on a cellular level (Parrish-Novak et al., 2015). A noteworthy case was reported by Predina et al., in which a complete osteosarcoma resection was performed under the real-time NIR imaging navigation with 5 mg/kg of indocyanine green (ICG) injection (Predina et al., 2018). In their follow-up report, Predina et al. documented a non-randomized, open-label study with all patients suspicious of osteosarcoma metastasis. Results indicated that bone tumors, which were difficult to be detected by traditional examination, were clearly visualized by ICG imaging, especially for the metastatic tumor with a size less than 1 cm. They further carried out a clinical trial with several sets of bone tumors to verify the preoperative NIR imaging-guided diagnosis and postoperative assessment of tumor status (Predina et al., 2019) (Figure 5). Although the employment of the NIR guidance system increased average surgical time, NIR bioimaging can clearly outline the anatomical structures preoperatively and intraoperatively, avoiding the unnecessary damage of nerve, blood vessel, lymph, and other adjacent tissues. In spite of the impressive output achieved, the injected doses of ICG were 10 times more than the standard clinical dosage for subcutaneous administration, which is common in the range between 0.1 and 0.5 mg/kg (Boni et al., 2015). In addition, another challenge is the translation of preoperative images into the patient’s physical anatomy in the clinical setting.

Bone metastasis of malignant tumors is the one of most crucial points that cause difficult treatment. A series of pathological processes occur during malignant tumor cells migrate to bone sites. These processes include tumor cells invasion and angiogenesis, tumor cells release into the vessels and/or lymphatics, implantation of tumor cells in the bone followed by their proliferation, and induction of osteolytic fracture (Li et al., 2014). It is essential to early detect and locate tiny bone metastasis to avoid further spread of tumor cells. Lim et al. synthesized a P800SO3 probe with inherent bone-targeting ability (Lim et al., 2017). The P800SO3 bioimaging clearly outlined ∼3-mm bone-metastasis tumor and distinguished the tumor margin from the surrounding normal sites. They further applied P800SO3 in the examination of the bone metastasis of breast cancer in the mice model (Lim et al., 2020). The metastatic tumor was fluorescently visualized in the bone marrow around the cortical bone in both the rib and proximal tibia. Odontogenic tumor is also a special type of intraosseous tumor. Morlandt applied IRDye800-labeled cetuximab antibody to target ameloblastoma *in vivo* and *in vitro* (Morlandt et al., 2020), showing higher SBR (2.35–4.12) than the IgG group (1.34–2.40). Collectively, we suggested the noninvasive NIR fluorescence could potentially be used as a diagnostic strategy for primary bone cancer and bone metastasis in the future clinic.

Although clinical trials of NIR fluorescence imaging have produced impressive results, the penetration depth is still limited. Photoacoustic imaging (PAI) is also a promising noninvasive imaging modality with maximum penetration depth over several centimeters. Spirou et al. have demonstrated that PAI is capable of sensitive detection of thermally induced changes at depths of up to 30–50 mm using *ex vivo* liver tissue (Spirou Orchard et al., 2004). Photoacoustic signal has been found to be sensitive to minor variations in cortical bone density (Lashkari and Mandelis, 2014). Further research suggested that photoacoustic imaging could differentiate cancellous and cortical bone without drilling or disrupting the pedicle, potentially allowing for spinal fusion surgery (Shubert and Lediju Bell, 2018). Jana Humbert et al. demonstrated that photoacoustic tomography monitoring of ICG-labeled liposomes in the murine bone marrow cavity provided an imaging depth over 4 mm (Humbert et al., 2020). The combination of PAI and fluorescence imaging will be the future direction in the diagnosis and assessment of bone metastases, targeted drug delivery, bone marrow-derived diseases, and bone physiology.

## PhotoX Therapy of Bone Cancer by NIR Probes

The clinical symptoms of primary bone tumors and bone metastases include pathological fracture, neurological compression, and skeletal-related events, bringing severe physical suffering to the patient. Besides surgical resection, chemotherapy and radiotherapy are widely applied in clinic, but drug resistance and considerable systemic side effects may result in tumor recurrence and endless suffering to patients (Ou and Guo, 2007). Therefore, NIR photoX therapies (photothermal, photodynamic, photoimmuno) have been developed as effective therapeutic methods, providing minimally invasive damage to normal tissues.

Photothermal therapy is on account of the photothermal effect of photothermal transduction agents (PTAs) that can transform light energy into heat so that locally damage the plasmalemma of tumor (Liu et al., 2019). Currently, NIR-responsive biomaterials have been extensively developed as PTT agents for osteosarcoma treatment and/or as bone-regenerating agents for bone repair, such as carbon dots (NIR-II) (Geng et al., 2020), multi-walled carbon nanotubes (NIR-II) (Lin et al., 2015), black phosphorus nanosheets (NIR-I) (Yang et al., 2018), graphene oxide (NIR-I) (Dang et al., 2018), copper-based chalcogenides (NIR-I) (Dang et al., 2018). Jiang et al. introduced the zoledronate as an extra targeting group while preserved the superparamagnetic iron oxide as the magnetic targeting group to enhance the osteosarcoma-targeting ability (Jiang et al., 2020). *In vivo* studies substantiated that dual-targeting groups could produce better local PTAs accumulation for PTT, significantly inhibiting the growth of breast cancer cells in the tibia. The PTT effectiveness is largely dependent on the penetration depth of applied lasers while bone tissue generally blocks the light, particularly in short wavelength. Liu et al. designed a type of graphene quantum dots (named 9T-GQD) with high-efficiency NIR-II light absorption to improve the penetration depth of PTT (Liu et al., 2020). The high photothermal conversion efficacy (33.45%) of 9T-GQD could significantly reduce the applied laser power. The tumor ablation by NIR-II PTT in the mice model was confirmed by histological examination. Of note, there was negligible damage to the surrounding normal tissues.

**FIGURE 6:**
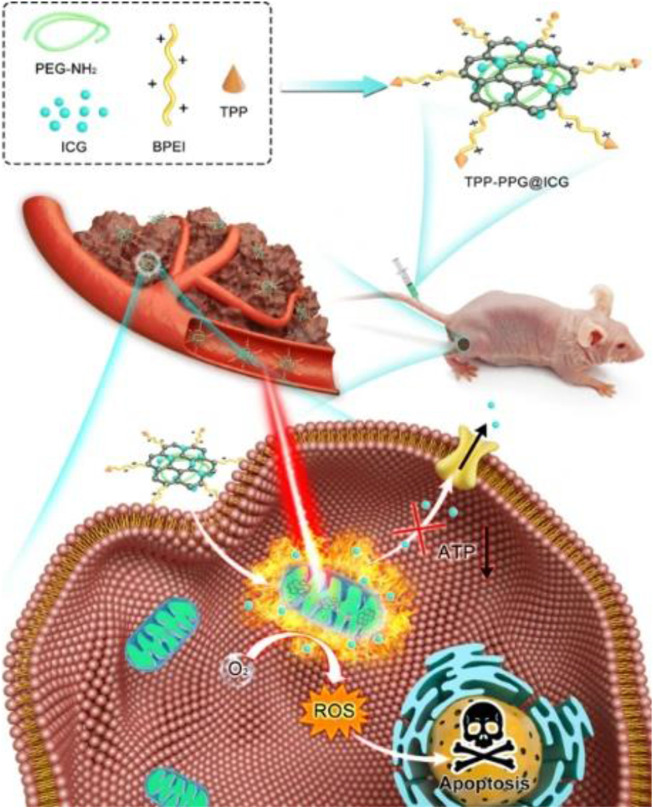
Schematic illustration showing TPP-PPG@ICG nanocomposite targeting mitochondrion for synergistic phototherapy. The PS agent (ICG) was grafted onto the PEG- and BPEI-functionalized photothermal agent (NGO) to obtain TPP-PPG@ICG. After cellular internalization, TPP-PPG@ICG accumulated in mitochondria, induced mitochondria-related intrinsic apoptosis, surmounted drug resistance, and enhanced the antitumor efficacy after 808-nm laser irradiation. Reproduced with permission from Zeng et al. (2021).

NIR photodynamic therapy (PDT) is a high-efficiency clinical treatment strategy for bone-related tumors with minimal side effects, high biosafety, and high controllability (Park et al., 2019). The effect of PDT relies on the concentration of photosensitizer (PS) at the treatment site, thus requiring a higher targeting ability to enhance the therapeutic efficiency and reduce the administrated PS dosage (Carina et al., 2019). Generally, the PS agents of PDT are efficient fluorophores, capable of both PDT and fluorescence bioimaging ability (Xu et al., 2018). Boron dipyrromethene (BODIPY), as a typical potential PS, has great advantages of intense absorption at a relatively long wavelength, long triplet excited-state lifetime, and excellent photostability (Sun et al., 2019). However, the application of BODIPY is limited by its hydrophobic rigid structure (Le et al., 2016). Zhu et al. synthesized an NIR triphenylamine-grafted BODIPY derivative (BDPTPA) by the nanoprecipitation approach, thus obtaining water-soluble and stable BDPTPA NPs with high singlet oxygen yield (35.2%) and a wide absorption range (600–1,100 nm) (Zhu J. et al., 2019). The as-prepared BDPTPA NPs enabled osteosarcoma ablation both *in vivo* and *in vitro*. Clinically available ICG is considered as an impressive NIR-I dye with PDT ability (Dash et al., 2021). Tsukanishi et al. designed ICG-loaded lactosomes for imaging-guided PDT (Tsukanishi et al., 2014). The deficiency of ICG-loaded nanocarriers was their long-term retention in the body circulation. Zeng et al. loaded the 4-carboxyl-butyl-triphenyl-phosphonium bromide (TPP, a mitochondria targeting group) and ICG in polyethylene imine-modified PEGylated nano-graphene oxide (GO), producing a nanoformula that could induce mitochondrial stress damage in tumor by NIR-I imaging-guided PDT (Zeng et al., 2021) (Figure 6). The nanoformula was fluorescently observed to accumulate in the tumor site 24 h post-administration, leading to the notable tumor (143b osteosarcoma) elimination with apoptosis and necrosis.

Combining PDT with PTT is a synergistic strategy for improving the treatment efficacy of cancers (de Melo-Diogo et al., 2017). PDT/PTT combination therapy was experimentally confirmed to be effective to bone tumors. In detail, membrane damage caused by PTT can enhance the effect of singlet oxygen produced by PDT in tumor by reducing the defense capability of the membrane system (Zhu J. et al., 2019). Despite of the remarkable progress, rare imaging-guided PDT was achieved in orthotopic bone tumors, given the penetration limitation of light. Future laboratory-scale and preclinical therapeutic efforts should comprehensively consider PTT/PDT/imaging combination agents to achieve an optimal diagnosis/therapeutic outcome.

Near-infrared photoimmunotherapy (NIR-PIT) is an emerging therapy strategy which uses a targeting antibody chemically conjugated with a photoabsorber/photosensitizer. The laser excitation could cause the locally targeted cancer cells to swell and burst, inducing necrotic cell death. The released contents from tumor cells could promote the activation of immune response and establish the long-term immunity for destroying tumor cells (Kobayashi and Choyke, 2019; Kobayashi et al., 2020). NIR-PIT has been successfully applied against brain tumors through AC133mAb conjugated with IR700, efficiently targeting the AC133 + glioblastoma stem cells, inducing the rapid tumor cell death and shrinkage of tumor in nude mice (Jing et al., 2016). Report has proved that NIR light (810 nm with 35.1 mW/cm^2^ power density) could transmit the bone with the maximum penetration thickness approximately 5 mm (Hart and Fitzgerald, 2016; Nakamura et al., 2019). Nakamura et al. evaluated the PIT efficacy through pan-IR700 probe on the A431-luc-bearing tumor mice covering a bovine rib, and the positive results indicated that the NIR-PIT was potential in bone cancer therapy (Nakamura et al., 2019).

## Perspective and Challenges

Numerous novel NIR fluorophores have been developed and evaluated in bone cancer because fluorescence imaging in the near-infrared window is a highly promising technique for biomedical applications with deeper tissue penetration capability and higher SBR (Zhu S. et al., 2019). However, there are still some issues that remain to be resolved before the probes can be used in clinical diagnosis. First, current bone tumor diagnostic probes mainly target minerals (e.g., hydroxyapatite), while the tumor cell–targeting probes are still lacking (Soodgupta et al., 2015). This situation leads to the failure of probe-related drug delivery and direct treatment of the bone tumor. As a result, it is essential to develop NIR fluorophores with high targeting ability to bone cancers, and exploiting bone cancer cell–targeting probes is likely critical to precisely decide the diagnosis/therapeutic efficiency. Second, probes with immunotherapy function play an indispensable role in cancer treatment (van der Meel et al., 2019). With the development of immumo/gene bone cancer therapy (Kager et al., 2017; Landi et al., 2019; Wang et al., 2020), the combination with photoX (e.g., thermal and dynamic) therapy is a promising option for bone cancer treatment. Third, new bone tumor targets need to be systematically screened. It is of great significance for clinicians to conduct early diagnosis, boundary determination, postoperative evaluation, and long-term follow-up using efficient imaging systems. Given the diversity at different stages of bone tumors, the next decade should enrich the library of targeting probes. The advances in NIR imaging modality need to be coupled with innovations in fluorophores and imaging systems, and future development on these points will provide promising options in the diagnosis and therapy of bone tumors.
